# A Rare Case of Non-puerperal Uterine Inversion Caused by a Prolapsed Fibroid Polyp

**DOI:** 10.7759/cureus.107554

**Published:** 2026-04-22

**Authors:** Dur-e- Shahwar, Samina Saleem

**Affiliations:** 1 Obstetrics and Gynecology, Patel Hospital, Karachi, PAK

**Keywords:** chronic uterine inversion, huge fibroid polyp, non-puerperal uterine inversion, prolapsed fibroid polyp, surgical management

## Abstract

Non-puerperal uterine inversion is an extremely rare gynecological condition associated with significant diagnostic challenges. A high index of suspicion is essential when evaluating patients with atypical gynecological presentations to ensure timely diagnosis. Although multiple etiologies have been reported, it is most commonly associated with a prolapsed endometrial polyp, submucosal fibroid, or, less frequently, a malignant mass. Imaging modalities, including transabdominal and transvaginal ultrasound, magnetic resonance imaging (MRI), and computed tomography (CT), play a crucial role in confirming the diagnosis and identifying the underlying cause. Consideration of the patient’s fertility is important, as uterine preservation may not be feasible in all cases. We, here, report the case of a 40-year-old nulliparous woman with a known diagnosis of fibroid uterus for the past three years, who presented with acute severe lower abdominal pain, urinary retention, and sudden prolapse of a large fibroid polyp measuring approximately 20 × 14 cm. Uterine inversion was suspected based on clinical examination and confirmed by radiological findings. Vaginal myomectomy was performed, and the uterine inversion was reduced manually under direct visualization. The patient had a smooth post-operative recovery.

## Introduction

Uterine inversion is a rare obstetric-gynecological emergency in which the uterus turns inside out, with the fundus collapsing downward into the uterine cavity and passing through the cervix or vagina. In obstetrics, uterine inversion usually occurs in the immediate postpartum period after the second stage of labor, with the uterine fundus descending alongside the placenta, whereas in gynecology, it is quite rare. Although relatively uncommon in puerperium, non-puerperal uterine prolapse is a very rare occurrence to the extent that there is no good estimate of its incidence [1]. Considering the rarity of the condition in the non-puerperal state, it is often difficult to diagnose; therefore, a high index of suspicion is needed to diagnose and manage [2].

Although there is no universally accepted classification system, uterine inversion is commonly categorized based on the degree of fundal descent. Stage I (incomplete inversion) occurs when the uterine fundus remains within the uterine cavity. Stage II (complete inversion) is characterized by the fundus passing through the cervical ring. Stage III (total inversion) involves protrusion of the fundus through the vulva, while Stage IV represents complete inversion of both the uterus and vagina, with both structures protruding beyond the vulva [3].

It may present acutely with something coming out of the vagina along with some bleeding or vaginal discharge; in other cases, it may be there for a long time and present late. Often, it is idiopathic, but it can be associated with other gynecological conditions such as benign uterine tumors in 70-80% of cases (leiomyoma, endometrial polyps), or malignant tumors in the remain­ing, especially in young women (leiomyosarcoma, mixed Müllerian sarcoma, rhabdomyosarcoma, endometrial and cervical carcinoma) [4,5].

The exact pathophysiology behind the uterine inversion is not known; however, the most common mechanism explains the attachment of the mass with the fundus, causing pressure due to its weight, leading to downward traction on the fundus and pulling the fundus downwards, hence putting pressure on the cervix, dilating the cervix, and inverting inside out in the vagina [6,7].

We, here, report a case of a nulliparous female with non-puerperal uterine inversion with a huge prolapsed fibroid. To our knowledge, this is the first case of non-puerperal uterine inversion with such a huge prolapsed fibroid uterus. We hope this case highlights the importance of early recognition and prompt management of rare clinical entities to prevent significant morbidity and improve patient outcomes, and underscores the need for a high index of suspicion when evaluating atypical presentations to avoid delays in diagnosis and treatment.

## Case presentation

A 40-year-old nulliparous woman, married for four years, with no known comorbidities, presented to the emergency department with a sudden protrusion of a mass per vagina. She reported an acute onset of severe lower abdominal pain three days prior, which was associated with urinary retention, necessitating urethral catheterization for the preceding three days. Her past medical and surgical history was unremarkable, except for multiple iron infusions administered over the last three years for anemia secondary to chronic menorrhagia. She had not previously sought gynecological evaluation for her heavy menstrual bleeding. Her menstrual cycles were regular, with heavy flow not associated with dysmenorrhea.

**Figure 1:**
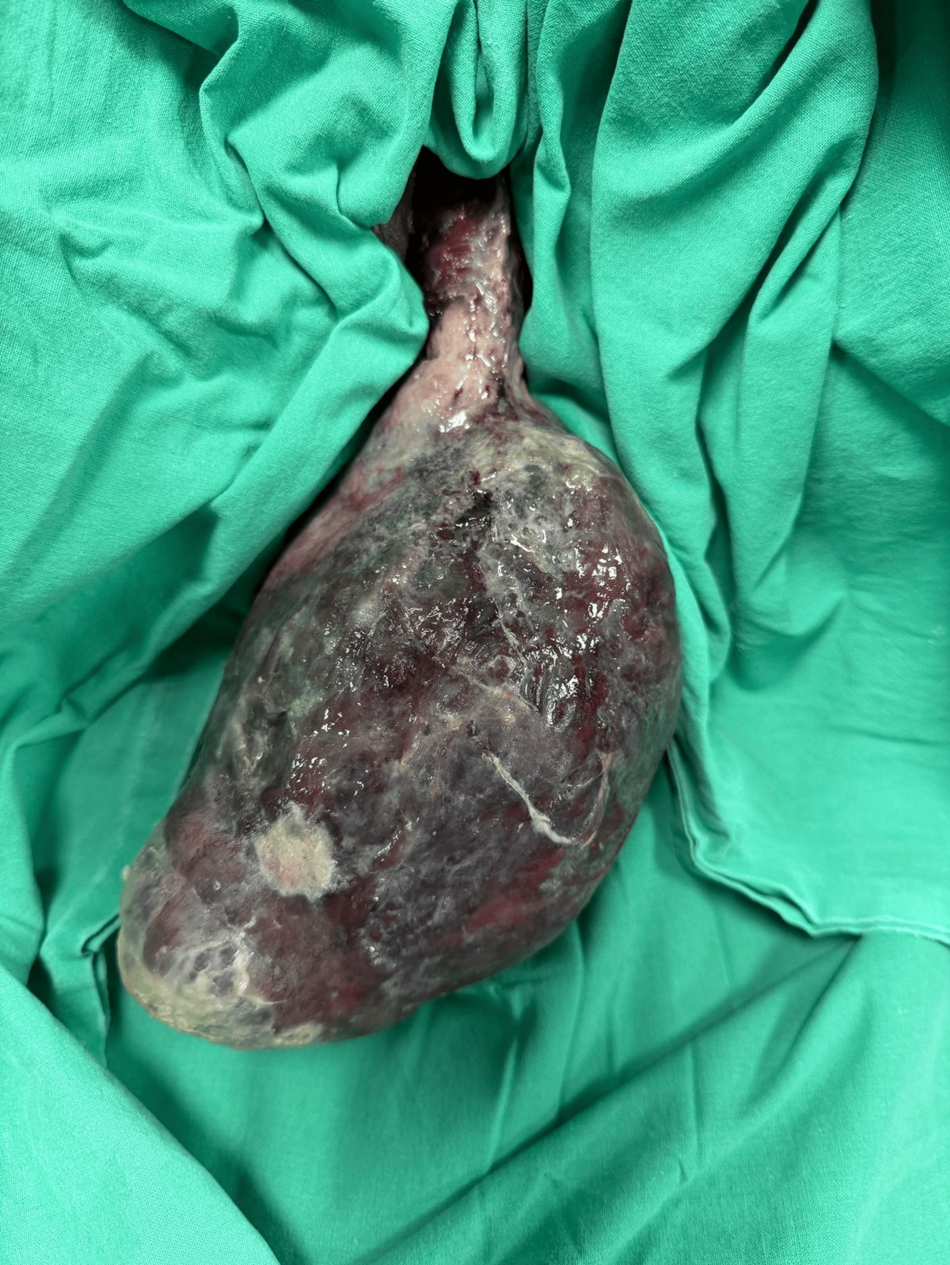
Fleshy necrotic mass seen hanging out of the vagina.

On examination, the patient appeared anxious and markedly pale. A Foley catheter was in situ. There was no icterus, cyanosis, clubbing, or koilonychia. Her vital signs were stable: blood pressure 107/84 mmHg, pulse rate 100 beats/min, temperature 98.6°F, and respiratory rate 24 breaths/min. Chest examination revealed bilateral equal air entry, and cardiovascular examination demonstrated normal heart sounds (S1 and S2). Abdominal examination revealed a soft, non-tender abdomen. Local examination showed a large, fleshy mass measuring approximately 20 × 14 cm, protruding outside the vagina and attached by a thick stalk, with minimal bleeding noted. On palpation, the mass was firm and heavy. On per vaginal examination, a firm, ring-like constriction was palpated within the vagina, suggestive of uterine inversion (Figure 1).

The patient was admitted to the ward. The protruding mass was washed with normal saline and wrapped in sterile gauze. Antibiotics were started, and a few investigations were sent, as shown in Table 1.

**Table 1:** Patient lab parameters. hpf: high-power field.

Parameter	Value	Reference range
Hemoglobin (Hb)	4.8 g/dL	12–16 g/dL
Total leukocyte count (TLC)	6.8 × 10³/µL	4.0–11.0 × 10³/µL
Platelet count	322 × 10³/µL	150–400 × 10³/µL
Ferritin	6 ng/mL	12–150 ng/mL
Urine white blood cells	3/hpf	0–5/hpf
Urine pus cells	Few	None
Urine culture and sensitivity	No growth	No growth

**Figure 2:**
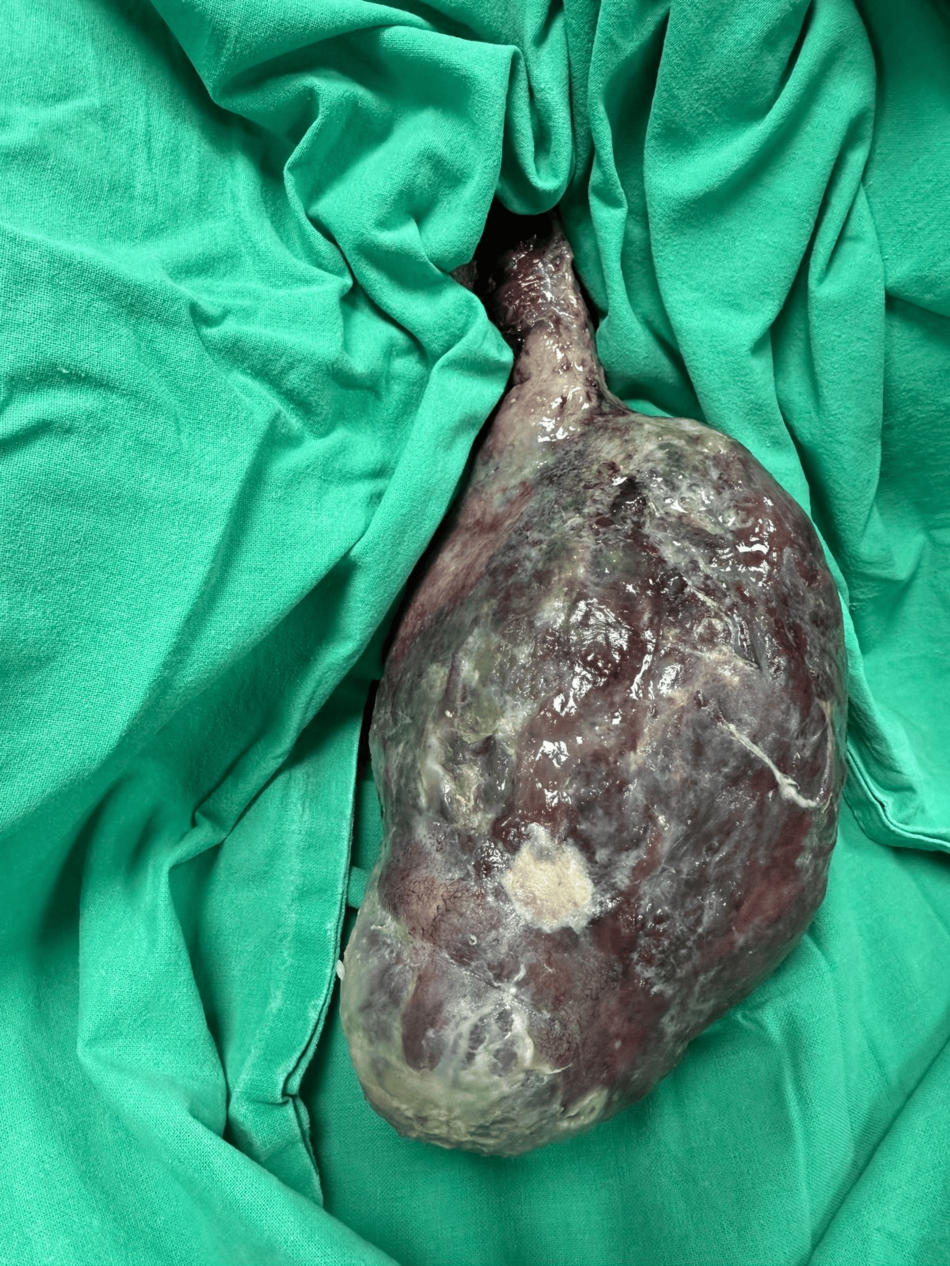
Prolapsed fibroid polyp with patchy necrotic areas.

Ultrasound was done, which showed complete uterine inversion. A multidisciplinary team approach was taken, involving a hematologist, an anesthetist, and a senior gynecologist. The patient was transfused with four packed cell volumes (PCVs) in four days; pre-op assessment was performed. Repeat CBC showed Hb 8.7 g/dl. Written informed consent was taken regarding the removal of the fibroid, hemorrhage, possible need for blood transfusion, and a 1-2% risk of hysterectomy. Patient was kept nil per oral from midnight 12 am and kept first on the list. On the day of surgery, a senior anesthetist was made available. The patient was given general anesthesia, and after endotracheal intubation, the patient was kept in the lithotomy position. After cleaning and draping the abdomen, the first examination under anesthesia was done. Mass was re-examined, found to be highly vascular with few necrotic patches attached via thick pedicle to inside the vagina, and no constriction ring was found (Figure 2).

**Figure 3:**
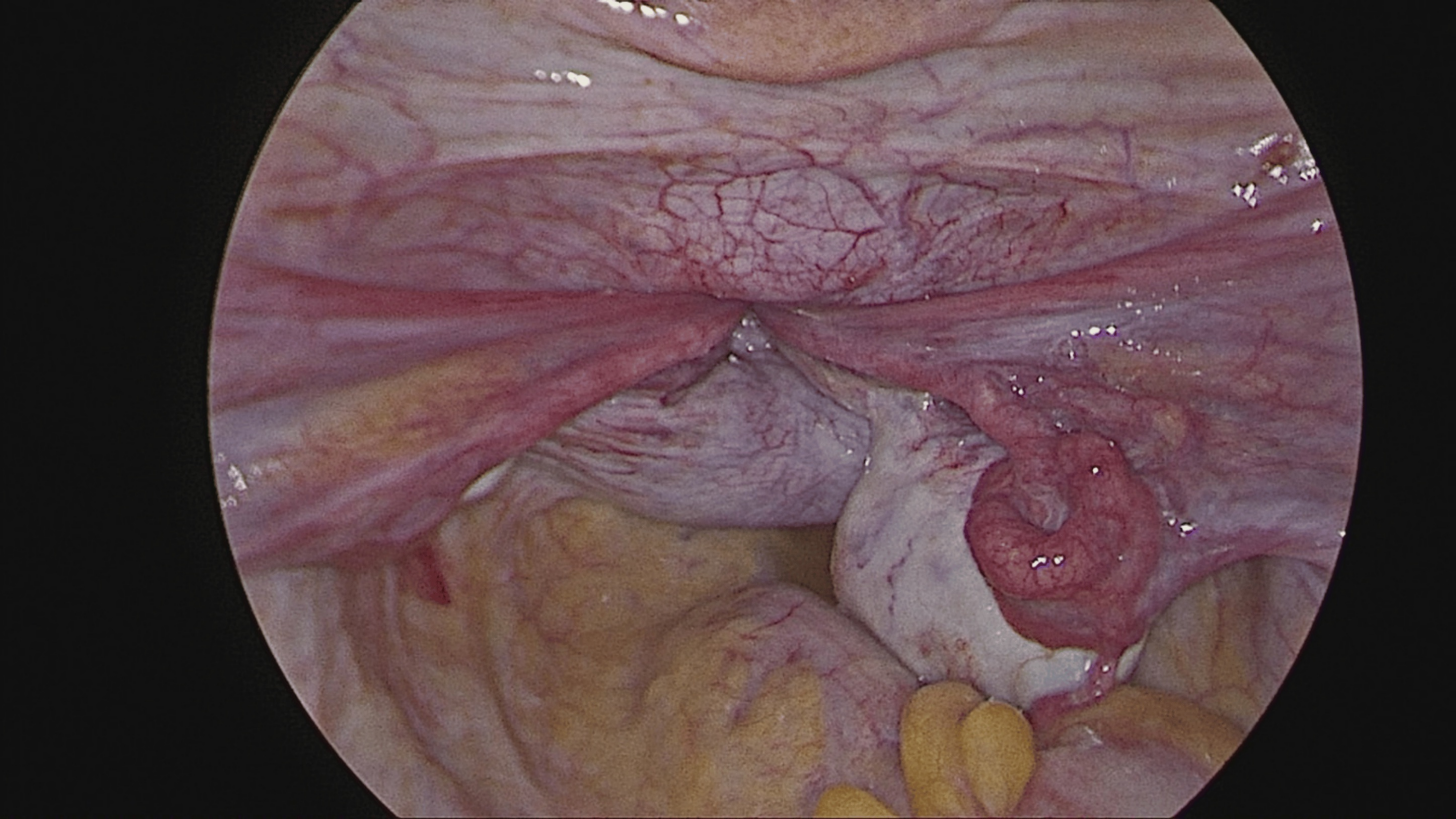
Laparoscopic view of uterine inversion. The laparoscopic image demonstrates a central concavity at the uterine fundus with inward traction of the round ligaments and fallopian tubes, consistent with uterine inversion. The constriction ring can be appreciated at the level of the cervix.

Laparoscopy proceeded, 10 mm primary port inserted via direct entry, pneumoperitoneum created, pelvis inspected, complete uterine inversion seen with both adnexa stretched (Figure 3).

**Figure 4:**
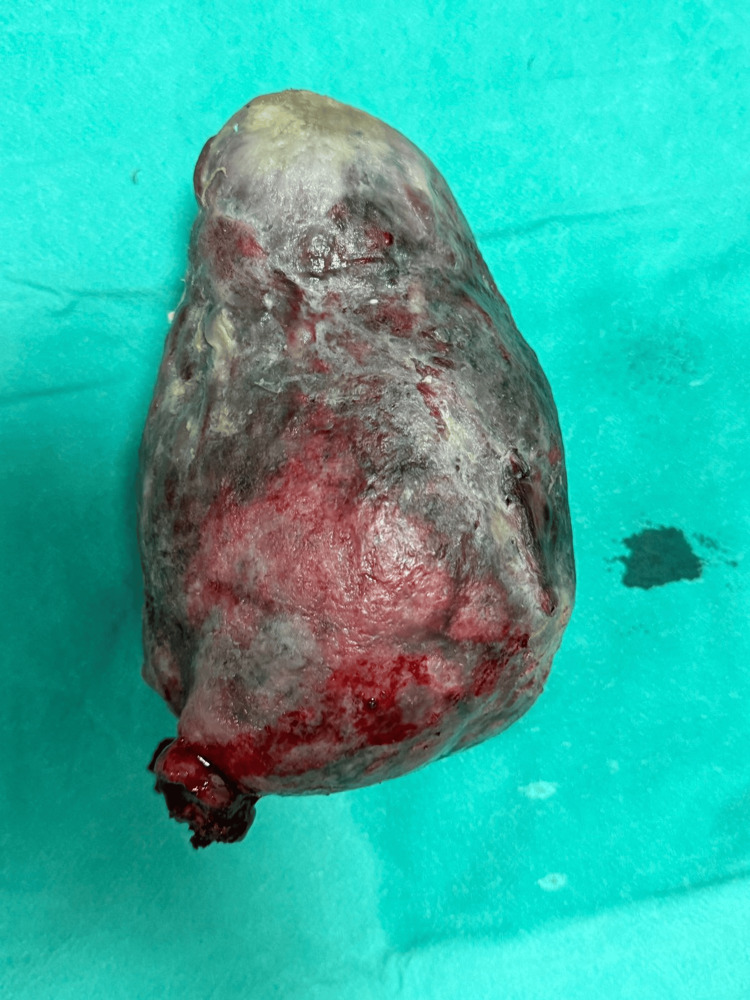
Gross specimen of the removed fibroid polyp following surgical excision. The excised specimen shows a pedunculated fibroid polyp with a smooth outer surface. Areas of congestion/necrosis may be noted.

Myomectomy proceeded vaginally by clamping and cutting the vascular pedicle of the fibroid polyp, and a sample was saved for histopathology (Figure 4).

**Figure 5:**
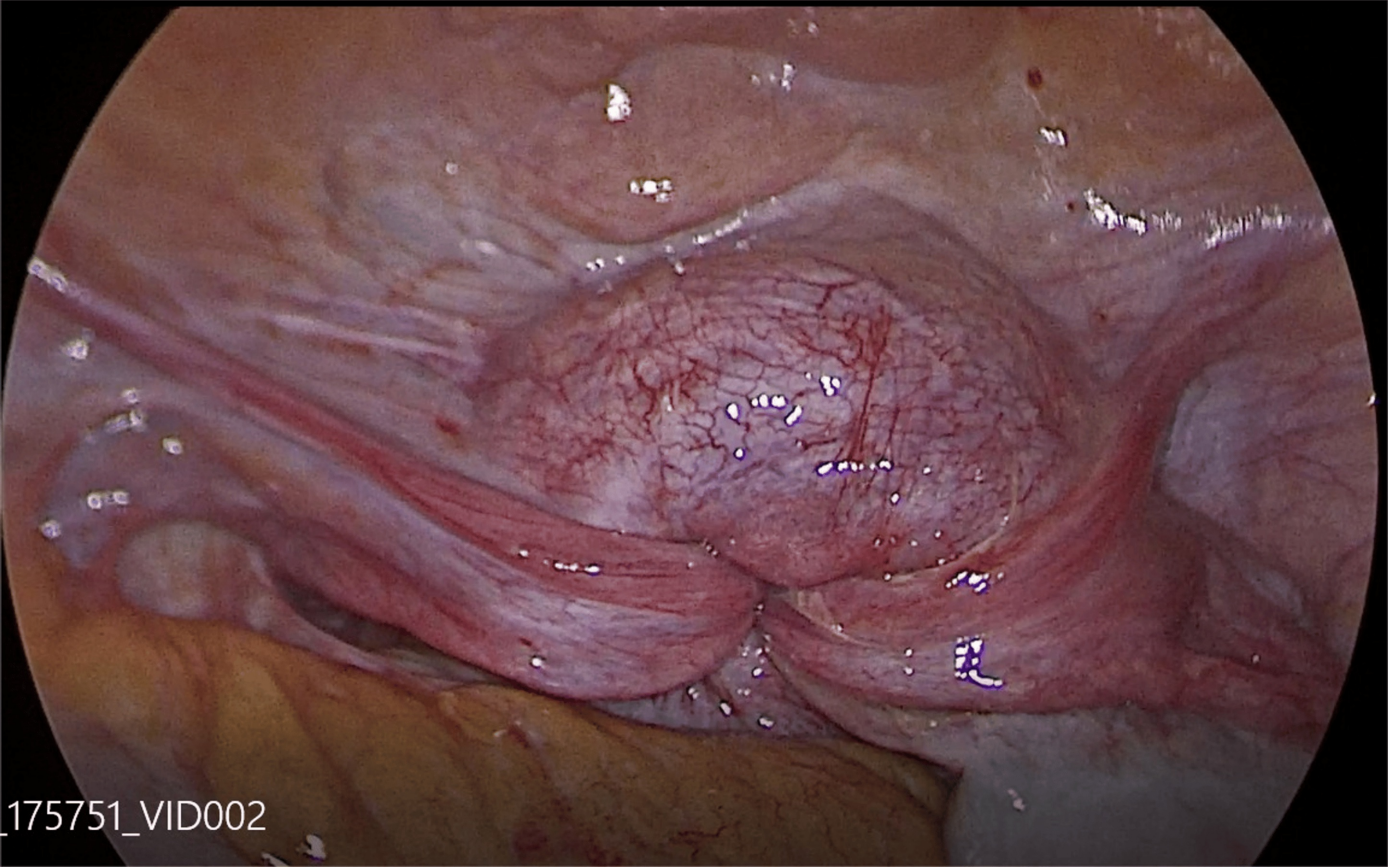
Intraoperative view demonstrating reduction of uterine inversion following surgical intervention.

**Figure 6:**
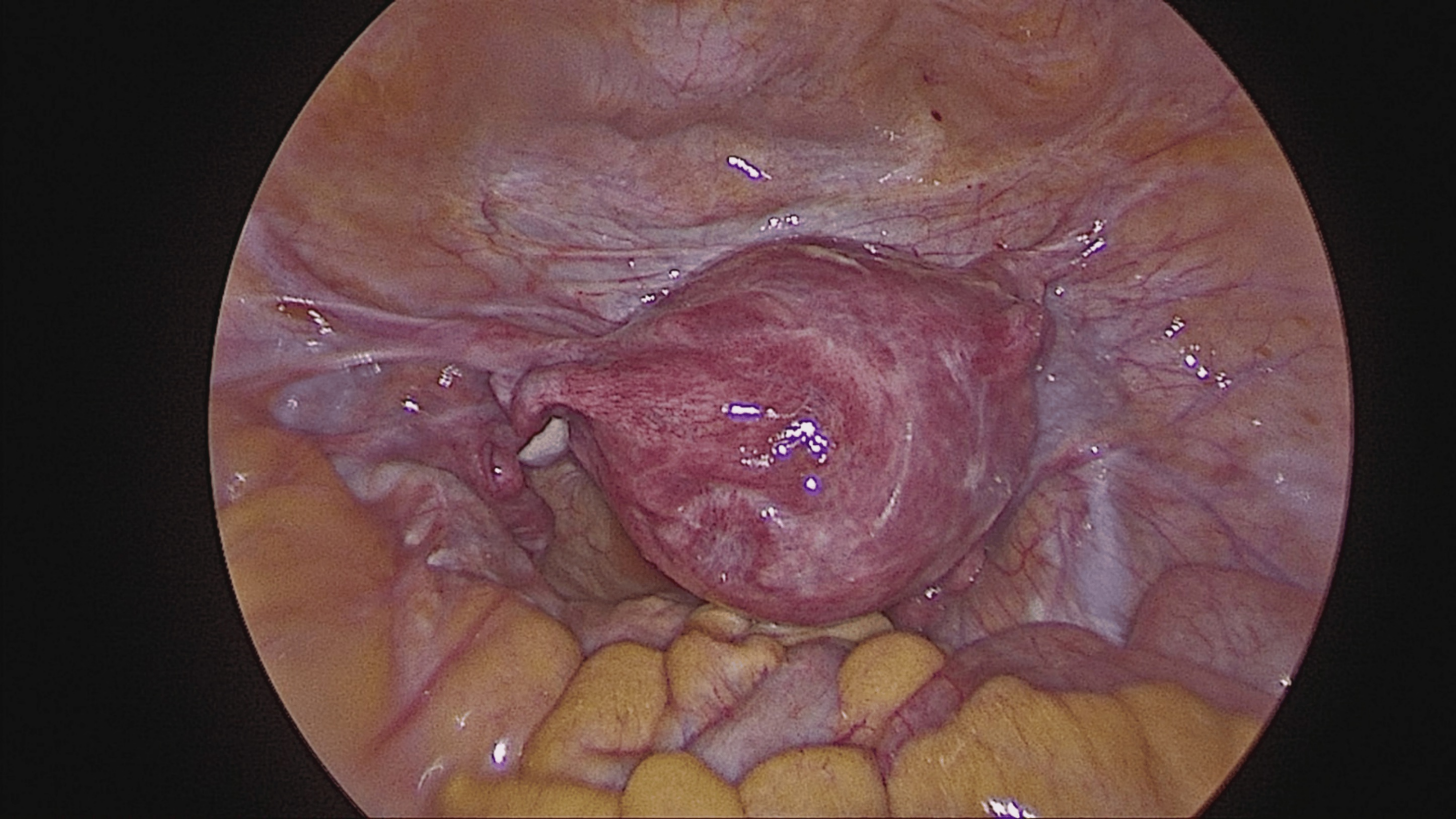
Intraoperative view at the end of the procedure. The image demonstrates successful reduction of uterine inversion with restoration of normal uterine anatomy. The uterine fundus is repositioned to its anatomical location, and the previously constricted cervical ring appears relaxed. The round ligaments and adnexal structures have returned to their normal orientation.

Per vaginal examination done and reduction attempted manually by Johnson's method, the uterine inversion was reduced completely under vision (Figures 5, 6). No bleeding observed, hysteroscopy done, uterine cavity visualized, no abnormality noted.

Patient remained vitally and clinically stable postoperatively; the Foley catheter was removed on the first post-operative day, and urinary retention was relieved. The patient was discharged on the second post-operative day after a dose of parenteral iron. The histopathology report showed leiomyomata.

## Discussion

Patients with puerperal uterine inversion present with shock, usually, while patients with non-puerperal uterine inversion often present with nonspecific symptoms such as vaginal mass, abnormal uterine bleeding, pelvic discomfort, or, in advanced cases, a mass protruding through the introitus. This can pose a diagnostic challenge, as it may mimic more common conditions such as prolapsed fibroid, uterine prolapse, or even malignancy. In our case, the patient presented with a huge mass protruding from the vagina, which is consistent with previously reported cases in the literature.

The diagnosis is primarily clinical but may be supported by imaging modalities, such as ultrasound or MRI, particularly in early or incomplete cases where findings may be subtle. Prompt recognition is crucial, as delayed diagnosis may increase the risk of complications including hemorrhage, infection, and shock [8,9].

Management depends on the severity of inversion, the patient’s hemodynamic status, and the desire for future fertility. Surgical intervention is often required in non-puerperal cases due to the presence of an underlying pathology. Various surgical techniques have been described, including abdominal approaches such as the Huntington and Haultain procedures, as well as vaginal approaches like the Spinelli and Kustner techniques [10]. In many cases, hysterectomy may be necessary, particularly when fertility preservation is not a concern or when malignancy cannot be excluded. In our patient, there was a huge prolapsed fibroid along with uterine inversion, for which myomectomy was done with manual reduction of uterine inversion under direct vision.

This case highlights the importance of maintaining a high index of suspicion for uterine inversion in women presenting with a vaginal mass, especially in the presence of a fundal fibroid. Early diagnosis and appropriate management are essential to prevent serious morbidity. Furthermore, reporting such rare cases contributes to the limited existing literature and aids in improving clinical awareness and decision-making.

## Conclusions

Non-puerperal uterine inversion remains a rare clinical entity, often associated with underlying uterine pathology such as submucosal fibroids. Its nonspecific presentation may lead to delayed diagnosis and increased risk of complications. This case underscores the importance of maintaining a high index of suspicion and demonstrates that timely surgical management can result in successful anatomical restoration and improved patient outcomes.
